# DIFFERENCES IN HOME HEALTH SERVICES AND OUTCOMES FOR MEDICARE ADVANTAGE PLANS WITH EPISODIC VS. PER-VISIT PAYMENTS

**DOI:** 10.1093/geroni/igae098.3835

**Published:** 2024-12-31

**Authors:** Rachel Prusynski, Anthony D’Alonzo, Michael Johnson

**Affiliations:** University of Washington, Seattle, Washington, United States; BAYADA Home Health Care, Pennsauken, New Jersey, United States; BAYADA Home Health Care, Pennsauken, New Jersey, United States

## Abstract

For millions of older adults receiving home health (HH) services annually, those with Medicare Advantage (MA) plans receive fewer visits and have worse functional outcomes compared to similar patients with Traditional Medicare (MA) plans. However, the thousands of MA plans available to beneficiaries are not created equal. MA plans with episodic payments have fewer administrative restrictions for HH providers than MA plans that pay per visit. This study of 285,297 HH episodes from 2019-2022 used robust inverse probability of treatment weighting to detect differences in HH service provision and patient outcomes between TM, episodic MA, and per-visit MA plans. Patients with both MA plans had shorter HH episodes and fewer visits from nurses, physical, occupational, and speech therapists, and home health aides than TM. Compared to TM, patients with episodic MA were less likely to improve in mobility and self-care function (Odds ratio [OR] 0.92 and 0.94, respectively) but had lower rates of inpatient admissions during HH (OR=0.95). Patients with per-visit MA had higher rates of community discharge (OR=1.06) and inpatient admissions (OR=1.06) than TM. Per-visit MA plans had higher rates (OR=1.12) of inpatient admissions than episodic MA. While both MA plans provide fewer HH services than TM, outcomes for MA plans versus TM are mixed depending on the MA plan payment structure. However, when comparing MA plans, per-visit plans have higher rates of inpatient admissions than episodic plans, a negative patient outcome. HH providers negotiating with MA plans should prioritize episodic payments to maximize outcomes and reduce administrative burden.

